# ^23^Na chemical shift imaging in the living rat brain using a chemical shift agent, Tm[DOTP]^5–^

**DOI:** 10.1007/s10334-022-01040-4

**Published:** 2022-09-02

**Authors:** Awais A Bajwa, Andreas Neubauer, Michael Schwerter, Lothar Schilling

**Affiliations:** 1 Division of Neurosurgical Research, Medical Faculty Mannheim, Heidelberg University, Mannheim, Germany; 2 Department of Computer Assisted Clinical Medicine, Medical Faculty Mannheim, Heidelberg University, Mannheim, Germany; 3 Institute of Neuroscience and Medicine (INM-4), Medical Imaging Physics, Forschungszentrum Jülich, Jülich, Germany; 4 European Center of Angioscience (ECAS), Medical Faculty Mannheim, Heidelberg University, Mannheim, Germany

**Keywords:** Magnetic resonance spectroscopy, Sodium, Chemical shift reagent, Blood–brain barrier, Rats

## Abstract

**Objective:**

It is well known that the use of shift reagents (SRs) in nuclear magnetic resonance (NMR) studies is substantially limited by an intact blood–brain barrier (BBB). The current study aims to develop a method enabling chemical shift imaging in the living rat brain under physiological conditions using an SR, Tm[DOTP]^5−^.

**Materials and methods:**

Hyperosmotic mannitol bolus injection followed by 60 min infusion of a Tm[DOTP]^5−^ containing solution was administered via a catheter inserted into an internal carotid artery. We monitored the homeostasis of physiological parameters, and we measured the thulium content in brain tissue *post mortem* using total reflection fluorescence spectroscopy (T-XRF). The alterations of the ^23^Na resonance spectrum were followed in a 9.4T small animal scanner.

**Results:**

Based on the T-XRF measurements, the thulium concentration was estimated at 2.3 ± 1.8 mM in the brain interstitial space. Spectroscopic imaging showed a split of the ^23^Na resonance peak which became visible 20 min after starting the infusion. Chemical shift imaging revealed a significant decrease of the initial intensity level to 0.915 ± 0.058 at the end of infusion.

**Conclusion:**

Our novel protocol showed bulk accumulation of Tm[DOTP]^5−^ thus enabling separation of the extra-/intracellular ^23^Na signal components in the living rat brain while maintaining physiological homeostasis.

## Introduction

Next to ^1^H, ^23^Na is the second most abundant nuclear magnetic resonance (NMR)-detectable nucleus in the body, although it has a markedly lower signal strength compared to ^1^H. Current sodium imaging studies present information on the total ^23^Na^+^ tissue content using ultra-high-field strength scanners, i.e., 7 or 9.4 Tesla (T) in patients [1–3] and even up to 21.1 T in experimental settings [4, 5]. However, beyond tissue content the extra-/intracellular concentration gradient is more relevant as it is involved in many basic cellular features including regulation of resting membrane potential, cellular osmolarity and volume, as well as in the generation of action potentials in excitable cells. Hence, delineation of the intra-/extracellular components of the ^23^Na signal is gaining increasing attention in NMR studies.

A widely used approach for the assessment of the extra-/intracellular Na^+^ ion distribution in experimental studies requires the application of shift reagents (SRs) which induce a characteristic shift of the ^23^Na resonance frequency upon interaction with sodium ions. All SRs are hydrophilic lanthanide-containing complexes carrying multiple negative charges. Upon systemic application, SRs readily distribute to most extracellular compartments, while they are barred from the intracellular space as long as the cell membrane is intact. Under these conditions, the intra- and extracellular ^23^Na signal components can be separated by their different frequencies as previously shown in experimental studies on heart and skeletal muscle, liver, and kidney in vitro [6–8] and in vivo [9–12].

Due to their physicochemical features, SRs cannot enter the brain parenchyma as long as the blood–brain barrier (BBB) is intact. In fact, the BBB, made up of the continuous endothelial lining of the cerebral blood vessels, is a major obstacle in many studies across the field of neuroscience [13]. Thus, the use of SRs requires methods to circumvent the BBB. A widely used one is based on bolus infusion of a hyperosmotic solution into the internal carotid artery (intracarotid [i.c.] infusion) leading to shrinkage of the endothelial cells and loosening of the intercellular junctions. This approach has been utilized in many experimental studies (for review, see Rapoport [14]) and applied in the clinical setting to facilitate the entrance of chemotherapeutics into the brain of tumor patients (e.g. Doolittle and coworkers [15]). The current study aims at developing a protocol employing hyperosmotic BBB opening to allow bulk accumulation of an SR, thulium(III)1,4,7,10 tetraazacyclododecane-N,Nʹ,Nʹʹ,Nʹʹʹ-tetramethylenephosphonate (Tm[DOTP]^5−^) and subsequent ^23^Na chemical shift imaging in the living rat brain.

## Material and methods

The study was approved by the local Animal Ethics Committee at the Regional Governmental Board (Regierungspraesidium Karlsruhe) and performed in accordance with the relevant national laws and institutional guidelines for the care and use of animals in research under the Directive 2010/63/EU and in compliance with the ARRIVE guidelines as introduced by Kilkenny and coworkers [16] meaning all efforts to minimize pain and stress for the animals and limit the number of animals used.

### Experimental groups

Male Sprague–Dawley rats were obtained from Janvier (Isle St. Genest, France). Before entering the experiment, animals were kept for at least one week under standard housing conditions with free access to rat chow and tap water.

Two series of experiments were performed. In the first series, the protocol for the hyperosmotic opening of the BBB was established and the effect of intracarotid (i.c.) infusion of Tm[DOTP]^5−^ (Macrocyclics, Dallas, USA) on physiological parameters studied. This included recording of arterial blood pressure, regional cerebral blood flow changes (by laser Doppler flowmetry [LDF]), and arterial blood homeostasis (arterial blood samples were drawn every 15 min and blood gases and plasma electrolyte levels were measured in a blood gas analyzer [Cobas b221, Roche, Mannheim, Germany]). In addition, accumulation of Tm in brain tissue samples was determined *post mortem*. In the second series, chemical shift imaging of the ^23^Na signal was performed in the scanner during i.c. infusion of Tm[DOTP]^5−^, here referred to as loading of the brain tissue.

A total of 20 animals were used. In the experiments in which Tm[DOTP]^5−^ was applied, 5 rats were excluded from the analysis due to a decrease of mean arterial blood pressure below 80 mmHg during loading of the tissue with the SR.

### Surgery

At the start of the experiments, the animals received a subcutaneous injection of atropine (Eifelfango, Bad Neuenahr, Germany; 40 µg/100 g body weight [bw]) to decrease tracheal secretion. A surgical level of anesthesia as characterized by the loss of nociceptive reflexes was established by an intraperitoneal injection of thiobutabarbital (Inactin™, Sigma-Aldrich, Taufkirchen, Germany; 15 mg/100 g bw) and maintained by fractional doses during the course of the experiments as necessary. Rectal temperature was measured by a thermistor coupled to a feedback-control heating table (Effenberger Geraetebau, Pfaffing, Germany) and body temperature maintained at 37 °C. Before commencing surgery, the eyes were covered by a layer of dexpanthenol ointment (Bepanthen^®^, Bayer, Leverkusen, Germany). Using an operation microscope (M650, Wild, Heerbrugg, Switzerland) animals were tracheotomized for controlled ventilation using a rodent respirator (Effenberger Geraetebau). A femoral artery and the accompanying vein were catheterized with polyethylene tubes (inner/outer diameter [i.d./o.d.], 0.58/0.96 mm). The arterial line served for recording arterial blood pressure using an Isotec pressure transducer (Hugo Sachs Electronics, March-Hugstetten, Germany) and drawing arterial blood samples. The venous line was used for saline infusion (0.1 ml/100 g bw/h) to balance any fluid loss. After ligating the left-sided external carotid and pterygopalatine artery a polyethylene tubing (i.d./o.d., 0.4/0.8 mm) filled with saline was introduced into the left common carotid artery and forwarded into the internal carotid artery (ICA) until the tip reached the skull base. In the animals, in experimental series 1 a burr hole was created over the left fronto-parietal cortex to study cerebral perfusion changes using an LDF monitor (DRT4, Moor, Axminster, England). Arterial blood pressure, body temperature, and the LDF signal were continuously fed into a home-built multichannel monitoring system based on LabView software (National Instruments, Munich, Germany) for online recording and offline analysis.

### Hyperosmotic opening of the BBB and subsequent loading of the brain tissue with Tm[DOTP]^5−^

Transient opening of the BBB was achieved by i.c. bolus infusion of a hyperosmotic (25%) mannitol solution. After dissolving the appropriate amount of mannit (Sigma-Aldrich) in a 20% mannitol stock solution (from Seray-Wiessner, Naila, Germany), the solution was warmed to 37 °C and passed through a filter (mesh size, 0.2 µm) before the infusion to prevent induction of microinfarcts as described previously [17]. The mannitol bolus (1.5 ml within 45 s) was immediately followed by i.c. infusion of an 80 mM Tm[DOTP]^5−^ solution (Tm[DOTP]^5−^ dissolved in a 220 mM NaCl solution to maintain osmolarity in the physiological range; infusion parameters, 0.1 ml/min for 60 min). All i.c. infusions were done using a laboratory syringe pump (LSP1, purchased from Hugo Sachs). We also performed control experiments with a normosmolar mannitol bolus (300 mM mannitol solution) followed by i.c. infusion of the 80 mM Tm[DOTP]^5−^ solution.

### Analysis of the tissue content of Tm

At the end of the 60 min loading period, 5% isoflurane (Forene™, Abbott, Wiesbaden, Germany) was added to the inspired gas mixture in some experiments in series 1. The abdomen was opened in the midline and the aorta exposed, distally ligated and cannulated with a wide bore tubing (i.d./o.d., 1.14/1.57 mm). After disruption of the caval vein, in situ perfusion with ice-cold physiological saline was started to flush the blood off the circulation. The brain was carefully removed, and cortical and subcortical tissue dissected from serial coronal sections. The tissues were collected for both hemispheres in pre-weighed tubes and after re-weighing stored at − 80 °C.

For analysis of the brain Tm content, the tissue samples were mixed with 50% trichloroacetic acid (from Merck, Darmstadt, Germany; ratio 1:6 *w*/*v*), carefully homogenized (Ultra Turaxx, IKA GmbH, Staufen, Germany) and centrifuged (10,000 rpm, 20 min, 4 °C; Biofuge Stratos, Heraeus, Hanau, Germany). The supernatant was removed and stored at − 80 °C for subsequent Tm measurement using the total reflection X-ray fluorescence analysis (T-XRF) methodology (S2 PicoFox, Bruker, Karlsruhe, Germany). The data obtained were converted into content per unit of wet weight. Based on these results, we estimated the interstitial concentration of Tm[DOTP]^5−^ assuming a 20% extracellular space fraction of the total brain volume [18].

### MRI scanning parameters

In the experiments of series 2, measurements were performed in a 9.4 T small animal NMR system (Biospec 94/20 USR, Bruker, Ettlingen, Germany). After finishing all surgical procedures as described above, the animals were positioned horizontally on a water-perfused warming blanket and body temperature was maintained using a feedback control system with an MR-compatible rectal probe. Blood pressure was monitored continuously, and an arterial blood sample was taken at the end of the experiment for blood gas analysis.

For ^23^Na imaging a homemade single loop surface coil (diameter, 15 mm) was positioned horizontally as close as possible to the head. Two Eppendorf tubes filled with saline were attached to the coil for off-line adjustment of ^23^Na and ^1^H images the latter being obtained by a 72 mm diameter commercial ^1^H birdcage resonator (Bruker).

First and second-order shim routines were performed on the ^23^Na frequency, followed by a pilot scout measurement on the proton resonance frequency with a three-dimensional T_2_ weighted RARE sequence to obtain a series of ^1^H images. The scanning parameters for the proton sequence were: repetition time/echo time (*T*_R_/*T*_E_), 2000/43 ms; matrix. 128 × 128 × 18; field of view (FoV), 37.5 × 24.0 × 30.0 mm^3^; resolution, 0.3 × 0.2 × 1.9 mm^3^; number of averages, 1, scan time per image, 160 s. A total of 8 coronal slices typically covered the brain from the frontal to the occipital pole.

Thereafter, the ^23^Na signal was recorded in the same slices. The first run was performed before bolus infusion (baseline conditions), followed by repeated runs during the Tm[DOTP]^5−^ infusion period. To obtain a spectrum in every single voxel, we used a Hanning weighted chemical shift imaging (CSI) sequence with three-dimensional data acquisition (3D CSI sequence) and free induction decay (FID) readout with these parameters: T_R_, 50 ms; acquisition delay T_AC_, 0.38 ms; unweighted matrix, 18 × 16 × 16; weighted matrix (after zero filling), 25 × 23 × 23 resulting in a total number of 13,225 voxels; FoV, 36 × 36 × 38 mm^3^; spectroscopic acquisition time, 40 ms; spectroscopic resolution, 12.5 Hz/point; nominal resolution, 2.00 × 2.25 × 2.36 mm^3^; reconstructed resolution, 1.4 × 1.6 × 1.7 mm^3^; number of averages, 4. Total acquisition time for each scan run encompassing all slices covering the brain was 10 min. Thus, a total of 7 data sets were obtained in each experiment.

### Processing of MRI data

Co-registration of ^23^Na images with the corresponding ^1^H images was performed by an in-house developed routine with the attached Eppendorf tubes serving as references. The ^23^Na images (resolution, 25 × 23 pixel) were upscaled to the 128 × 128 pixel resolution of the ^1^H images. Upscaling resulted in a distortion adding to errors introduced by a small rotation of the ^23^Na images and a slight mismatch in the *z* position. These errors were corrected by affine transformations until good alignment is achieved. The co-registered images were used to define the areas of both hemispheres in the coronal slices as regions of interest, ensuring that all ^23^Na measurements were taken from brain tissue only.

All CSI displays were reconstructed offline using the spatially encoded FIDs. The magnitude image was obtained by taking the average of the first 20 time points of each FID. Spatial encoding of spectroscopic information was obtained by clustering the voxels of the entire brain hemispheres, i.e., the manually defined regions of interest (see above) via Fourier transformation along the temporal dimension. To suppress higher order phase shifts, all spectra are presented in magnitude mode. Chemical shifts were calculated with respect to the resonance frequency obtained for the first scanning period (i.e. before loading of the brain with the SR was started).

### Phantom measurements with Tm[DOTP]^5−^

Increasing concentrations of Tm[DOTP]^5−^ (0, 0.1, 0.2, 0.5, 1.0, 2.0, 5.0 and 10.0 mM) were mixed with 5% agarose (made up in physiological saline solution) and studied in the 9.4 T scanner using a commercially available double tuned ^1^H/^23^Na volume resonator (Bruker) to achieve homogeneous excitation. The changes in the longitudinal (*T*_1_) and transverse (*T*_2_) relaxation times were measured and evaluated by applying global inversion recovery and spin echo sequences. The *T*_1_ and *T*_2_ relaxation time constants were extracted by nonlinear least-squares fit procedures. Moreover, a calibration curve to determine the chemical shift (δ) with respect to the pure agarose phantom sample (0 mM Tm[DOTP]^5−^) was constructed. These data were used for estimating the tissue concentration of the SR based on the chemical shift measured in vivo.

### Calculations and statistics

Calculations of MRI-based data including image co-registration were done using in-house developed software routines written in MATLAB (R2013a, The MathWorks, Natick, USA). Comparison of means was performed using either a t-test or the analysis of variance (ANOVA) procedure followed by the Fisher LSD test for pairwise post-hoc testing. All values in the text and tables are given as mean ± standard deviation.

## Results

*Experimental series 1: Recording of physiological parameters during loading with the SR and measurement of Tm accumulation in brain tissue.*

**Fig. 1:**
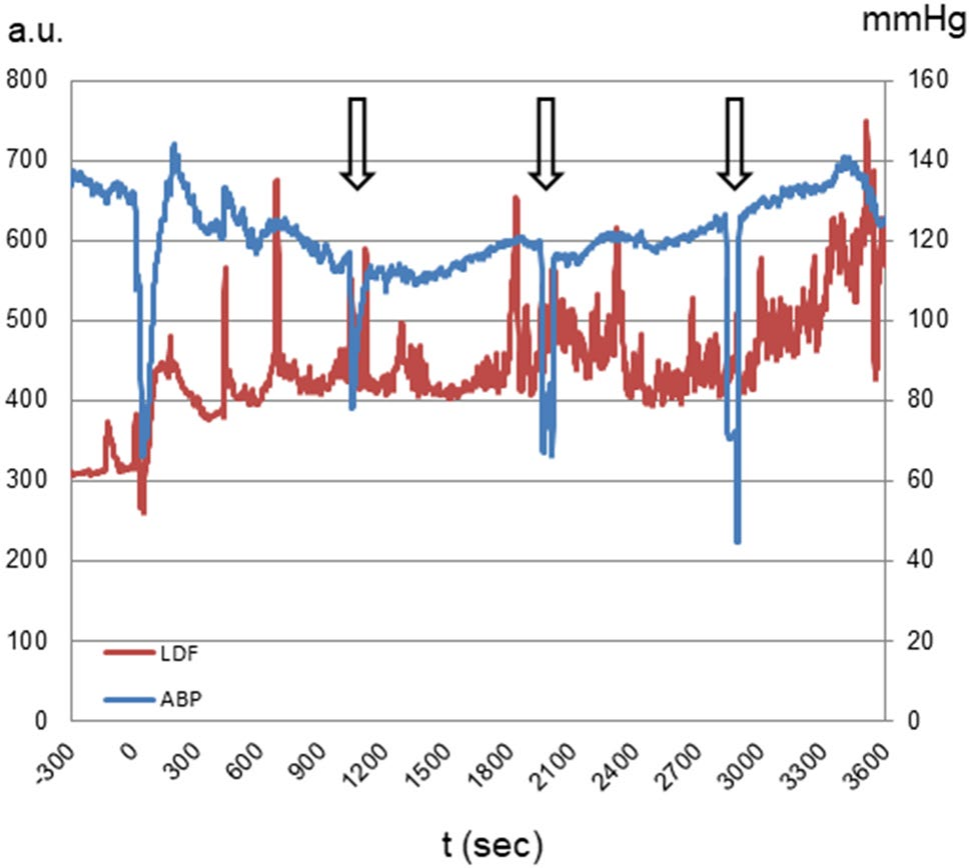
Effect of brain loading with intracarotid (i.c.) infusion of Tm[DOTP]^5−^ (80 mM, 0.1 ml/100 g body weight for 60 min) on arterial blood pressure (ABP in mmHg) and regional cerebral blood flow measured by laser Doppler flowmetry (LDF in arbitrary units [a.u.]) in the ipsilateral fronto-parietal cortex. 0 indicates the start of the hyperosmotic bolus injection (25% mannitol, 1.5 ml within 45 s) which was immediately followed by switching to Tm[DOTP].^5−^ infusion. Arrows indicate episodes of arterial blood sampling (the last sample was taken after stop of recording)

**Fig. 2:**
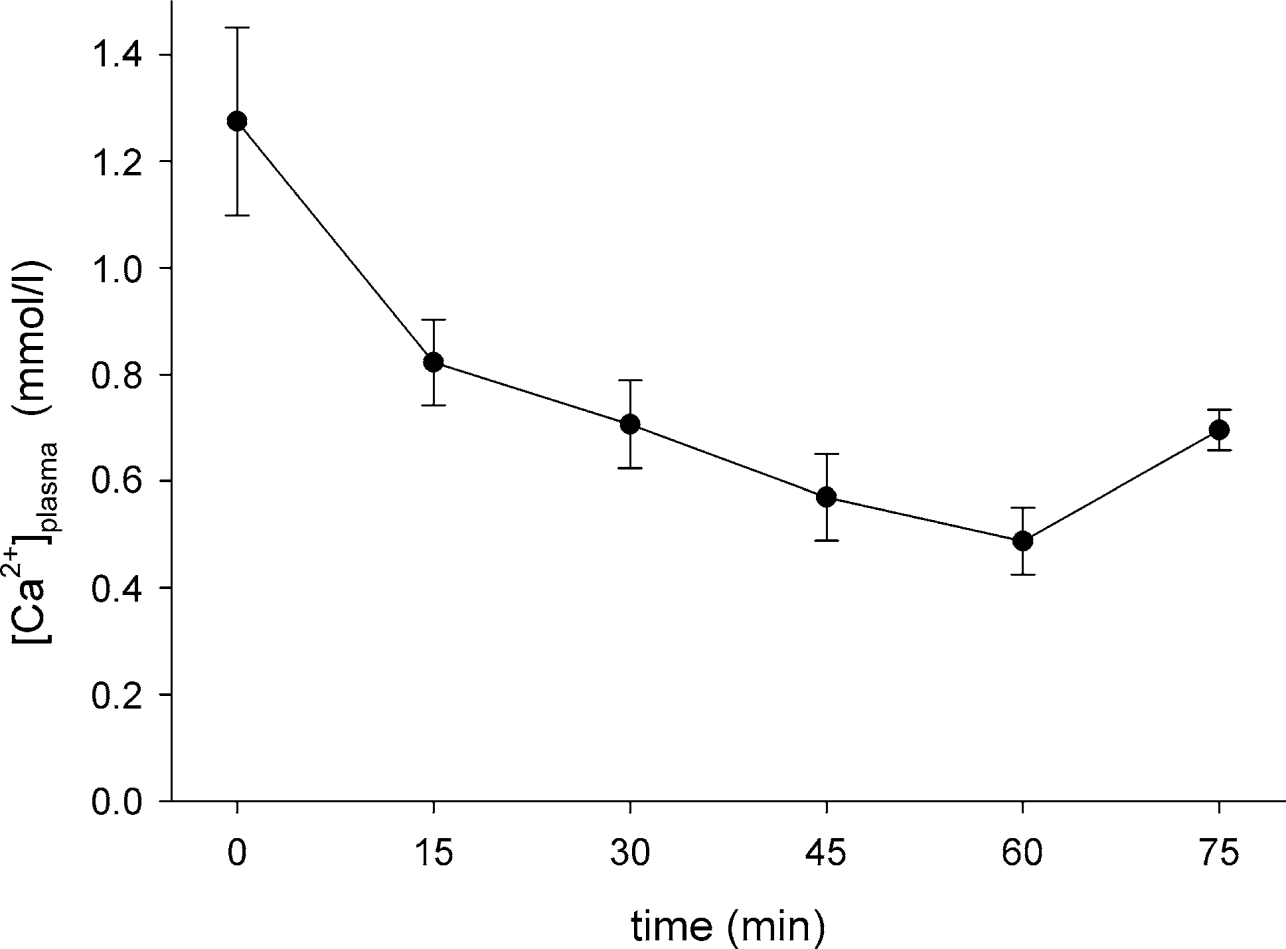
Alterations of the free plasma Ca^2+^ concentration measured every 15 min during intracarotid infusion of the shift reagent (SR), Tm[DOTP]^5−^ (80 mM solution, 0.1 ml/min, 60 min) following the hyperosmotic opening of the blood–brain barrier. The value given at *t* = 0 indicates the basal concentration. The time-related decrease of the Ca^2+^ ion concentration is probably due to complex formation with the SR. Note the partial recovery after cessation of the infusion (at *t* = 60 min). Given is mean ± standard deviation

To enable bulk accumulation of Tm[DOTP]^5−^ in brain tissue, an i.c. bolus infusion of a hyperosmolar (25%) mannitol solution was applied followed by infusion of the Tm[DOTP]^5−^ solution, again using the i.c. line. During the 60 min loading period, a slight to a moderate decrease of arterial blood pressure quite often occurred. This is illustrated in (Fig. 1) depicting the course of the arterial blood pressure along with the changes in LDF blood flow measured in the ipsilateral fronto-parietal cortex. If arterial pressure dropped to values below 80 mmHg, which was considered the lower threshold of cerebral autoregulation [19, 20], the experiment was aborted. Arterial blood samples, taken every 15 min, revealed virtually unchanged values for pH, pCO_2_, and pO_2_ as well as physiological Na^+^ and K^+^ concentrations. The results obtained before and at the end of the loading period are shown in (Table 1). However, there was a time-related decrease in the free Ca^2+^ concentration as shown in (Fig. 2). This decrease might well account for the hypotonic response described above.

**Table 1:** Blood gas analyses and electrolyte concentrations in arterial blood samples taken before and at the end of the 60 min Tm[DOTP]^5−^ loading period

	pH	pCO_2_ (mmHg)	pO_2_ (mmHg)	Na^+^ (mmol/l)	K^+^ (mmol/l)
Before loading	7.46 ± 0.06	39.2 ± 6.3	142.8 ± 40.7	144.5 ± 2.7	4.6 ± 0.5
After loading	7.44 ± 0.10	37.6 ± 9.9	192.8 ± 25.0	149.4 ± 4.2	3.8 ± 0.2*

Given are mean ± standard deviation

*p < 0.05 vs the respective value measured before loading

The Tm content in brain tissue samples taken *post mortem* was determined by means of T-XRF methodology. The results shown in (Table 2) indicate a marked accumulation of Tm in both, cortical and subcortical brain tissue in the ipsilateral hemisphere. Based on the Tm content obtained in µg/g wet weight the concentration of the SR in the interstitial fluid was estimated assuming a volume fraction of 20% according to Sykova and Nicholson [18]. In the contralateral hemisphere, Tm was also detectable although at a considerably lower level (Table 2). In control experiments, the estimated interstitial Tm concentration was < 100 µM in the ipsilateral hemispheric tissue samples, well in line with the fact that the SR does not readily cross the intact BBB.

**Table 2:** *Post mortem* measurement of the accumulation of thulium (Tm) in brain tissue after intracarotid (i.c.) bolus application of a 25% mannitol solution (1.5 ml in 45 s) followed by i.c. infusion of a 80 mM Tm[DOTP]^5−^ solution (0.1 ml/min for 60 min)

Tm[DOTP]^5−^	Hemispheric brain tissue			
	Ipsilateral		Contralateral	
	Cortical	Subcortical	Cortical	Subcortical
µg/g ww	407.8 ± 165	355.6 ± 213	104 ± 33.2	169.3 ± 46.5
mM (ECS)	2.23 ± 0.90^a^	1.95 ± 1.17^b^	0.57 ± 0.18	0.93 ± 0.26

The tissue content of Tm, obtained in µg/g wet weight (ww]) by total reflection X-ray fluorescence analysis (T-XRF) methodology was transferred into estimated mM concentrations in the extracellular space (ECS) assuming a 20% total brain volume fraction [18]. The results are given as mean ± standard deviation.

^a^*p* < 0.05

^b^*p* < 0.1 compared to contralateral tissue samples

### Experimental series 2: measurements in the scanner

**Fig. 3:**
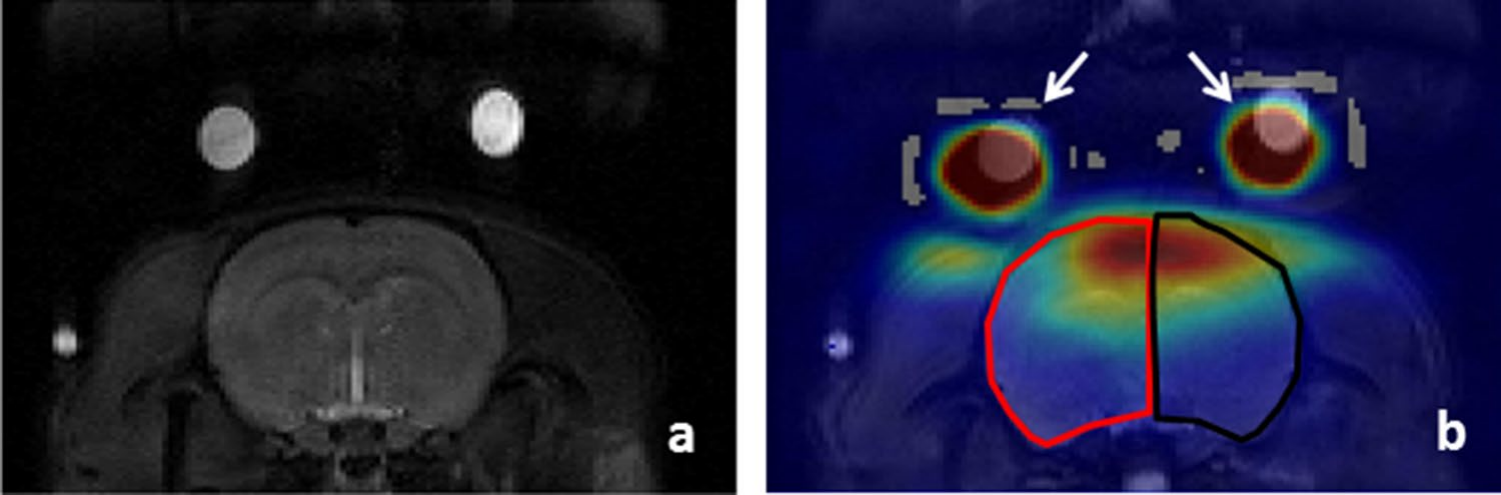
Exemplifying the co-registration of coronal slices of a rat’s head to allow measurement of the ^23^Na signal in brain tissue selectively. Depicted are the ^1^H images (**a**) and the ^23^Na image (**b**). The hemispheric areas are outlined in the ^1^H image and transferred onto the ^23^Na signal image with the red line indicating the ipsilateral and the black line the contralateral hemisphere (**b**). White arrows indicate the saline-filled Eppendorf tubes serving as references for co-registration. The images were taken during baseline conditions, i.e., before the hyperosmotic opening of the blood–brain barrier and loading of the brain tissue with the shift reagent, Tm[DOTP]^5−^

The ^23^Na images displayed a somewhat elliptic area of high signal intensity over the head reflecting the sensitivity profile of the ^23^Na surface coil. Co-registration of the ^1^H and the corresponding ^23^Na images allowed identification of the brain tissue to be analyzed selectively as shown in (Fig. 3).

**Fig. 4:**
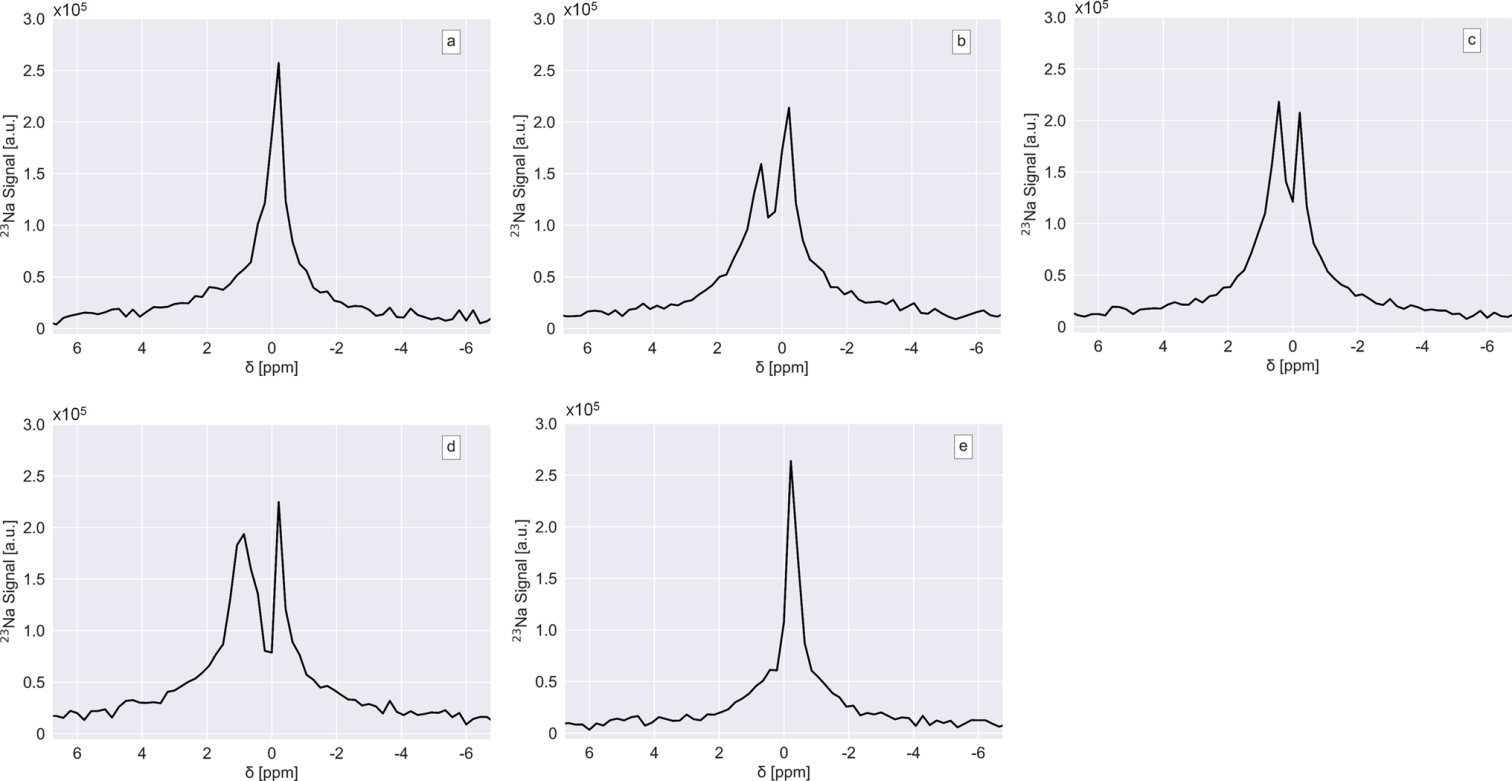
Spectroscopic analysis of the ^23^Na signal shown for the coronal brain slice depicted in Fig. 3. The chemical shift δ (given in ppm) is indicated on the *x*-axis and the signal intensity (given in arbitrary units) on the y-axis. The graphs were obtained in the ipsilateral hemisphere under baseline conditions (**a**) and after the hyperosmotic opening of the blood–brain barrier with a subsequent intracarotid infusion of Tm[DOTP]^5−^ (80 mM solution, 0.1 ml/min) for 20 min (**b**), 40 min (**c**), and 60 min (**d**). Figure 4e shows the ^23^Na signal in the contralateral hemisphere at the end of the 60 min loading period. The slight deviation of the peak at 0 ppm is probably due to small imperfections in the local shimming

Spectroscopic analysis showed a single small-based peak in the control period throughout all experiments. The frequency of this resonance peak, ω_0_, was 105.9 MHz (*δ* = 0 ppm) as expected from the 9.4 T field strength used. During the i.c. infusion of Tm[DOTP]^5−^ this peak eventually displayed a split in each slice of the infused hemisphere in all experiments with hyperosmotic BBB opening. This split typically appeared around 20 min after initiating loading with the SR and remained detectable until the end of the experiment. The spectroscopic analysis was performed following averaging the magnitude signal of all voxels with at least 20% of the maximum intensity (to improve SNR) in a given hemispheric slice. The appearance of the resonance spectrum and its alterations in the experimental course are exemplified in (Fig. 4) with the results obtained from the slice shown above (Fig. 3). In this case, the shift of the distinct second peak amounted to 1.5 ± 0.23 ppm resulting in an estimated interstitial Tm[DOTP]^5−^ concentration of 2.3 ± 0.35 mM based on the calibration curves obtained in phantom studies described below. In the contralateral, i.e. non-perfused hemisphere, we did not observe a peak split in any of our experiments. We also performed control experiments using a normo- instead of a hyperosmolar mannitol bolus. Under these conditions, there were no alterations of the ^23^Na resonance spectrum, most notably no indication of a curve split at all.

**Fig. 5:**
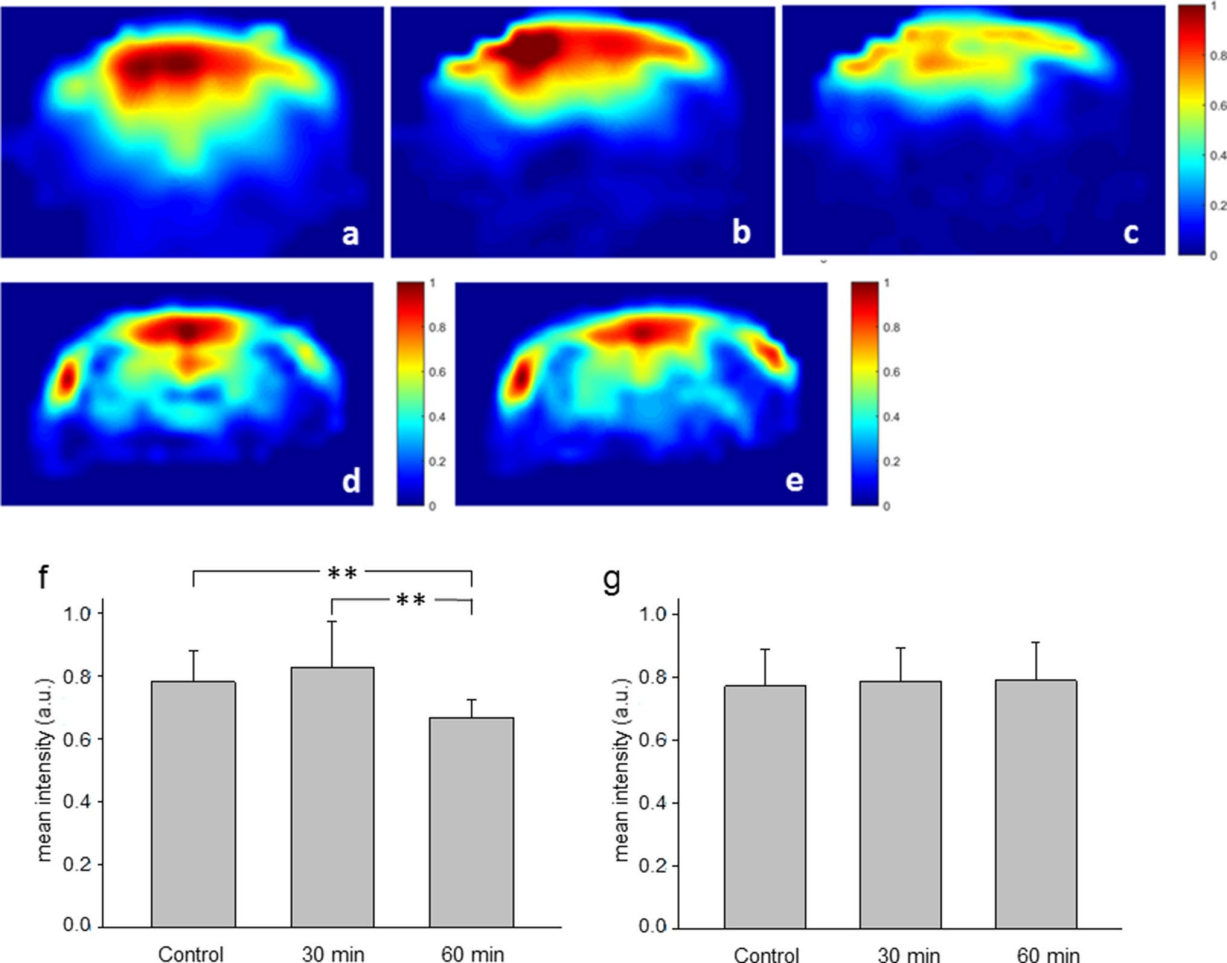
^23^Na CSI magnitude images at different time points during intracarotid (i.c.) infusion of Tm[DOTP]^5−^. Upper row: images obtained in an experiment with the hyperosmotic opening of the blood–brain barrier followed by brain loading with Tm[DOTP]^5−^ for 30 min (**b**) and 60 min (**c**). Middle row: images obtained in an experiment with a bolus of a normosmotic mannitol solution followed by infusion of Tm[DOTP].^5−^ for 60 min (**e**). All intensities are normalized with respect to the images obtained under baseline conditions, i.e., before mannitol bolus infusion (**a**, **d**). Bottom row: mean signal intensities obtained in the hemispheric slices shown in the upper row (**f**) and in the middle row (**g**). Given are mean values along with the standard deviation. **p < 0.01

We also studied the alterations of the signal intensities in the ^23^Na magnitude CSI images during i.c. infusion of Tm[DOTP]^5−^. Typical results are shown in (Fig. 5) (for an experiment with the hyperosmotic opening of the BBB in a, b, c, and f; for a control experiment in d, e, and g). The overall intensity did not display any apparent alteration in the first 30 min of the loading phase, but thereafter, gradually decreased until the end of the loading period in experiments with BBB opening (Fig. 5b, c). Quantitative analysis of CSI intensities over all slices of the hemispheres revealed a statistically significant reduction of intensity at the end of the experiment (Fig. 5f). In control experiments signal intensity did not display any striking change during the entire course of the experiment as exemplified in the images shown in Fig. 5 (baseline situation) and 5e (after 60 min of SR infusion). The results of the quantitative analysis are depicted in (Fig. 5g).

We tried to quantify the sodium concentrations in the intra- and extracellular space in individual slices by integrating the spectra of the two resonance peaks obtained at the end of the loading period. A voxel-by-voxel analysis did not work out due to an insufficient SNR in the majority of voxels. Therefore, we clustered all voxels in a given slice and applied a fit approach composed of two Lorentzian functions (*L*1, *L*2):

1 $$L1\, = \,{a \mathord{\left/ {\vphantom {a {\left( {\left( {b^{\Lambda } 2 - \omega \_0^{\Lambda } 2} \right)^{\Lambda } 2\, + \,\left( {c^{\Lambda } 2*\omega \_0^{\Lambda } 2} \right)} \right)}}} \right. \kern-\nulldelimiterspace} {\left( {\left( {b^{\Lambda } 2 - \omega \_0^{\Lambda } 2} \right)^{\Lambda } 2\, + \,\left( {c^{\Lambda } 2*\omega \_0^{\Lambda } 2} \right)} \right)}}$$

With a: amplitude, b: shift of the ^23^Na resonance frequency, ω_0: ^23^Na resonance frequency, c: width parameter

2 $$L\_2\, = \,{{\left( {1 - a} \right)} \mathord{\left/ {\vphantom {{\left( {1 - a} \right)} {\left( {\left( {d^{\Lambda } 2 - \omega \_0^{\Lambda } 2} \right)^{\Lambda } 2\, + \,\left( {e^{\Lambda } 2 * \omega \_0^{\Lambda } 2} \right)} \right)}}} \right. \kern-\nulldelimiterspace} {\left( {\left( {d^{\Lambda } 2 - \omega \_0^{\Lambda } 2} \right)^{\Lambda } 2\, + \,\left( {e^{\Lambda } 2 * \omega \_0^{\Lambda } 2} \right)} \right)}}$$

With a: amplitude, d: shift of the ^23^Na resonance frequency; ω_0: ^23^Na resonance frequency, e: width parameter, and

3 $$S\, = \,L1\, + \,L2\, + \,f$$

With S: superposition of L1 and L2, f: constant offset.

For the individual parameters, fitting procedures revealed values associated with outnumbering error ranges only. Therefore, we did not consider these results reliable indicating that the intra-/extracellular sodium concentrations cannot be estimated from the current data, unfortunately.

### Phantom measurements

**Fig. 6:**
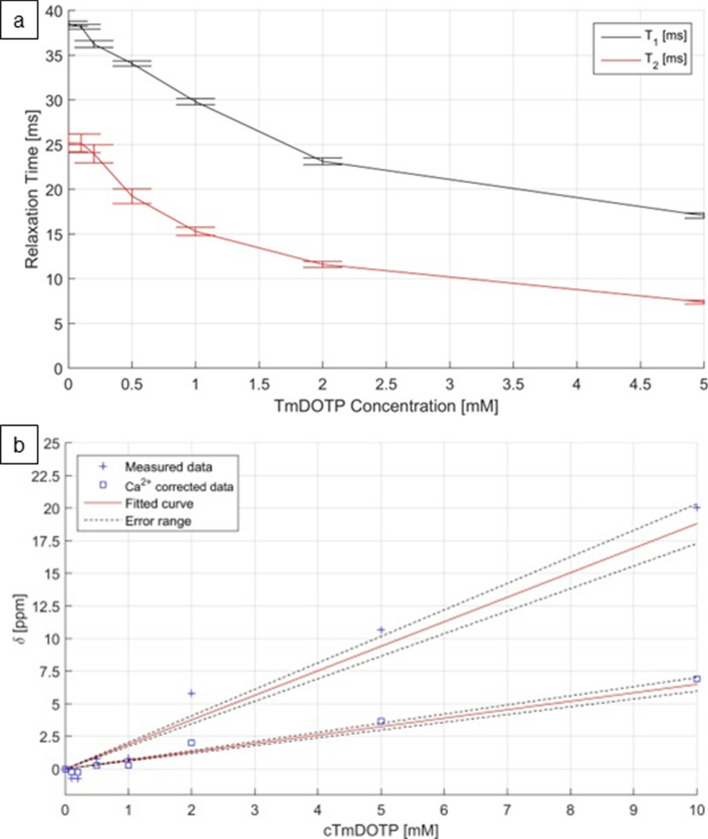
Phantom measurements to show the relationship between the concentration of the shift reagent, Tm[DOTP]^5−^ and the relaxation times T_1_ and T_2_ **a** and the shift (δ) of the ^23^Na signal **b** obtained in the 9.4 T NMR scanner. Tm[DOTP].^5−^ was dissolved in agarose gel (5%, made up in physiological saline solution). The values obtained for δ were adapted to in vivo conditions according to the method described by Puckeridge and coworkers [21]

We performed measurements of phantoms containing different concentrations of Tm[DOTP]^5−^ in a 5% agarose gel made up of physiological saline. The longitudinal (*T*_1_) and transverse (*T*_2_) relaxation times decreased with increasing concentrations of the SR, and this effect was somewhat more pronounced for T_2_ in the low concentration range (Fig. 6a). The linear change of the chemical shift induced by Tm[DOTP]^5−^ is shown in (Fig. 6b). Since the shift is affected by the Ca^2+^ concentration and by the temperature, the values obtained for the chemical shift in the phantom measurements were adapted to the in vivo conditions by introducing correction factors for these parameters as proposed by Puckeridge and coworkers [21]. This calibration curve was used to estimate the concentration of Tm[DOTP]^5−^ in the interstitial space as described above.

## Discussion

In MRI studies of the brain use of SRs such as Tm[DOTP]^5−^ is severely hampered in the presence of a functionally intact BBB, which effectively prevents the transit of SRs into brain tissue. A previous study in rats revealed for a dysprosium containing SR, Dy[TTHA]^3−^ a transfer constant 4 times less than that for sucrose, a known poor permeant at the intact BBB [22]. Nevertheless, there is still a contention concerning the use of SRs. Bansal and coworkers [23] and Ronen and Kim [24] described a resonance shift over the head of rats following intravenous (i.v.) infusion of Tm[DOTP]^5−^ without manipulation of BBB function. The authors deemed that this shift originated from the muscle tissue around the skull; thus emphasizing the importance of coregistration of ^1^H and ^23^Na images as part of our protocol. Although not yielding a perfect match, the coregistration routine clearly allowed to define the hemispheric areas within the ^23^Na images.

Reports from the Hyder group claimed that SRs such as Tm[DOTP]^5−^ can easily enter the brain tissue, suggesting that entry occurred either via passive diffusion over the operative BBB or via extravasation from the leaky blood vessels in the circumventricular organs [25–27]. The former is in conflict with the results of the present study and with data from the literature mentioned above. The functional importance of the latter pathway is questionable since the total surface area of these vessels is small, and the circumventricular organs (as well as the choroid plexus) are surrounded by ependymal cells with tight intercellular junctions to prevent the diffusion of blood-borne substances into the brain as reviewed elsewhere [28].

Recently, microapplication of Tm[DOTP]^5−^ into brain parenchyma has been reported to result in a small resonance shift [29]. However, the disturbance of the BBB function, either transient or permanent seems to be more efficient to allow SRs passing into the brain interstitial space. A hyperosmotic bolus enhanced the transfer constant of Dy[TTHA]^3−^ approximately 60 fold in rats [22], and it allowed the bulk accumulation of SRs as shown in the present study and in a previous one described by Eleff and coworkers [30]. According to their experimental protocol in dogs systemic administration of Dy[TTHA]^3−^ resulted in a large resonance shift of apparently intravascular origin, and it was only after bilateral i.c. bolus administration of a 25% mannitol solution that a separation between the extra- and intracellular compartment became visible.

In the present study, accumulation of the SR was measured in brain tissue samples taken *post mortem* by means of NMR-independent T-XRF methodology. This analytical method has been previously used to detect a wide range of trace elements including the rare earth element gadolinium in a variety of biological samples including brain tissue homogenates (including brain) as reviewed elsewhere [31]. The reliability of T-XRF-based measurements has been shown by using as reference methods atomic absorption spectrometry and inductively coupled plasma mass spectroscopy [32, 33] (for review, see also Szoboszlai and coworkers [34]). We found a high Tm content in brain tissue samples after hyperosmotic BBB opening, making up a mean value of 408 µg/ wet weight in cortical tissue samples (Table 2). This value corresponds to a Tm[DOTP]^5−^ concentration of 0.36 mmol/kg wet weight, which by chance matches data measured by Huang and coworkers in experimental glioma tissue samples (0.36 mmol/kg wet weight) using an NMR-based methodology [35]. It is well known that tumor vessels are leaky, thus lacking a barrier function. In addition, a markedly lower concentration of Tm[DOTP]^5−^, 0.13 mmol/kg wet weight was measured in peritumoral normal tissue [35], presumably due to spread from the intratumoral site of extravasation.

The concentration of Tm[DOTP]^5−^ at the BBB site builds up the driving force for diffusion into the brain. Huang and coworkers reported a concentration of approximately 5 mM in animals with bilateral renal artery ligation [35]. Using i.c. infusion of Tm[DOTP]^5−^ we can only estimate its concentration at the BBB site. In view of the 0.1 ml/min infusion speed and assuming an average cerebral blood flow of 120 ml 100 g^−1^ tissue min^−1^ with a hemispheric wet weight of 800 mg in rats [36] one may expect a roughly 1:10 dilution resulting in an effective concentration of approximately 8 mM Tm[DOTP]^5−^. This high driving force goes along with a much lower concentration of Tm[DOTP]^5−^ in the systemic circulation due to dilution upon drainage into the vena cava and continuous excretion by preserving renal function. Nevertheless, there must have been some residual accumulation of Tm[DOTP]^5−^ present as indicated by the decrease of the free plasma Ca^2+^ ion concentration (Fig. 2). This decline, most probably caused by complexation with the SR, may well explain the moderate decrease of arterial blood pressure observed in a couple of our experiments.

In each experiment with the hyperosmotic opening of the BBB, accumulation of Tm[DOTP]^5−^ in brain tissue eventually shifted the ^23^Na spectrum in the perfused hemisphere resulting in a peak split. The split appeared approximately 20 min after starting the SR infusion, and it persisted until the experiments were terminated. In fact, our recording protocol was designed to provide a reasonable temporal resolution at the expense of spatial and spectroscopic resolution. Unfortunately, the SNR did not allow a voxel-by-voxel analysis of the spectroscopic data. Clustering of voxels did not lead to improvement, which was most probably caused by phase shifts of higher order resulting in an annihilation of the signal when calculating the average of several voxels. Therefore, we performed a Fourier transformation of the magnitude signal averaging all voxels with at least 20% of the maximum image intensity in each hemisphere of a given slice. Whenever a peak split appeared, the unshifted peak was considered to reflect the intracellular ^23^Na signal component and the shifted peak to come from extracellular sources. We did never observe a third peak, presumably representing the intravascular compartment as reported previously reported by Eleff et al. [30] and by Naritomi and coworkers [12]. In these studies, Dy[TTHA]^3−^ was employed with a much higher systemic loading intensity (by a factor of four to ten on a molar basis) as compared to our regimen.

Chemical shift imaging during loading of the brain tissue with Tm[DOTP]^5^ showed marked alterations in the signal intensity. In phantom measurements shortening of T_1_ and T_2_ relaxation times was clearly related to the concentration of the SR (Fig. 6a). In low concentrations (≤ 0.5 mM Tm[DOTP]^5−^) the effect on T_1_ outweighed the effect on T_2_ mathematically. Although this may slightly increase the signal intensity, we did not observe a clear-cut effect in the early phase of tissue loading in vivo. When the concentration of the SR exceeds 0.5 mM in the phantom, T_2_ shortening became more prominent. Accordingly, the overall signal intensity decreased in the second half of the loading period in the hemispheres which had undergone hyperosmotic BBB opening (Fig. 5c, f).

Using the curves constructed on the results of the phantom measurements, we determined the interstitial Tm[DOTP]^5−^ concentration at the end of the loading period. The range of values was in fairly good agreement with those obtained *post mortem* using T-XRF measurements. However, these values could not be directly related to each other, because the data produced by T-XRF measurements were average values for cortical and subcortical tissue taken from the whole hemisphere, while data derived from the resonance shifts referred to individual coronal slices.

We tried to quantify extra- and intracellular sodium concentrations from the spectroscopic data using a fit composed of two Lorentzian functions but did not obtain reliable fit results. Potential reasons to explain this failure might include the poor SNR and the limited spatial resolution which did not allow the elimination of large blood vessels and cerebrospinal fluid-containing compartments since we had to consider the entire area of each coronal slice (as discussed above). Moreover, despite a fairly high concentration level of Tm[DOTP]^5−^ established in the brain tissue, it may not have been fully balanced with the ventricular and subarachnoid fluid during the course of the experiments preventing interaction of the SR with all sodium ions in the extracellular space. Similarly, Khan and coworkers [37] also failed to present absolute data, despite a considerably better SNR and higher spectroscopic resolution in their study.

The current study describes a novel protocol to establish a robust and reproducible shift of the ^23^Na resonance signal in the healthy rat brain with rigid control of physiological homeostasis. We quantified the accumulation of Tm[DOTP]^5−^ in brain tissue using an NMR-independent method and showed a clear connection between changes in T_1_, T_2_ and T_2*_ relaxation times and the concentration of the SR. Based on these results the subsequent experiments will focus on recording data with a high spatial and spectroscopic resolution. Loading of the brain tissue may be further enhanced by increasing the concentration of Tm[DOTP]^5−^ in the i.c. infusion solution. Furthermore, a microballoon catheter will be introduced into the epidural space of the contralateral hemisphere as described by Bendszus and coworkers [38]. This will provide an internal reference, which might be a useful tool for the quantification of concentrations as discussed elsewhere [37]. Still, the experimental approach as described in the present study may be applied for monitoring alterations of ^23^Na distribution in brain tissue under defined conditions, e.g. in the early phase of (regional or global) ischemia after loading with Tm[DOTP]^5−^.
